# Effects of L-carnitine supplementation on lipid profiles in patients with coronary artery disease

**DOI:** 10.1186/s12944-016-0277-5

**Published:** 2016-06-17

**Authors:** Bor-Jen Lee, Jun-Shuo Lin, Yi-Chin Lin, Ping-Ting Lin

**Affiliations:** The Intensive Care Unit, Taichung Veterans General Hospital, Taichung, 40705 Taiwan; Department of Nutrition, Sinying Hospital, Ministry of Health and Welfare, Tainan, 73042 Taiwan; School of Nutrition, Chung Shan Medical University, Taichung, 40201 Taiwan; Department of Nutrition, Chung Shan Medical University Hospital, Taichung, 40201 Taiwan

**Keywords:** L-carnitine supplement, Lipid profiles, Antioxidant, Coronary artery disease

## Abstract

**Background:**

L-carnitine (LC) plays an important physiologic role in lipid metabolism. To date, no clinical study has been performed to examine the effect of LC supplementation on the lipid status of coronary artery disease (CAD) patients. The aim of this study was to investigate the lipid lowering effects of LC supplementation (1000 mg/d) in CAD patients.

**Methods:**

CAD patients were identified by cardiac catheterization as having at least 50 % stenosis of one major coronary artery. Forty-seven subjects were recruited and randomly assigned to the placebo (*n* = 24) and to the LC (*n* = 23) groups. The intervention was administered for 12 weeks. The levels of LC, lipid profiles, and antioxidant enzyme activity (superoxide dismutase, SOD) were measured.

**Results:**

The subjects in the LC group had significantly higher SOD activity (20.7 ± 4.2 versus 13.1 ± 2.9 U/mg of protein, *P* < 0.01), high density lipoprotein-cholesterol (1.34 ± 0.42 vs. 1.16 ± 0.24 mmol/L, HDL-C, *P* = 0.03), and apolipoprotein-A1 (Apo-A1, 1.24 ± 0.18 vs. 1.12 ± 0.13 g/L, *P* = 0.02) than those in the placebo group at week 12. Triglyceride (TG) level was slightly significantly reduced (1.40 ± 0.74 vs. 1.35 ± 0.62 mmol/L, *P* = 0.06) and the level of LC was negatively correlated with TG and apolipoprotein-B (Apo-B), and positively correlated with HDL-C and Apo-A1 after LC supplementation. Additionally, SOD activity was significantly negatively correlated with lipid profiles (total cholesterol, TG, and Apo-B) after supplementation.

**Conclusion:**

LC supplementation at a dose of 1000 mg/d showed significantly increased in HDL-C and Apo-A1 levels and a slight decrease in TG levels but no other changes in other lipids in CAD patients, and this lipid-lowering effect may be related to its antioxidant ability. Further studies should be conducted to define an optimal dose of LC for lipid-lowering in patients with CAD.

**Trial registration:**

Clinical Trials.gov Identifier: NCT01819701

## Background

L-carnitine (LC) plays an important physiologic role in lipid metabolism. LC may carry long-chain fatty acids across the inner mitochondrial membrane for β-oxidation and adenosine triphosphate (ATP) production [1, 2]. Recent studies have demonstrated that LC is not only a transporter of lipids but is also an antioxidant [3, 4]. LC appears to interfere with the formation of reactive oxygen species, through its ferrous ion metal-chelating ability, thus stabilizing the free radicals formed on the α-carbon by conjugation [4, 5]. In addition to its antioxidant activity, LC also exhibits anti-inflammatory properties [6–8]. LC may inhibit the nuclear factor-kappa B pathway through the suppression of reactive oxygen species formation [9–11]. Whereas coronary artery disease (CAD) is associated with higher levels of oxidative stress and inflammation and hyperlipidemic status [12], it may be helpful to administer LC to CAD patients given its physiological functions.

A number of clinical studies have been conducted to examine the lipid-lowering effects of LC supplementation in patients with renal disease [7, 8, 12–17], diabetes [18–20], or hyperlipidemia [21, 22], however, the results regarding the lipid-lowering effects of LC supplementation are inconsistent due to different disease statuses. To date, no clinical study has been performed to examine the effect of LC supplementation on the lipid status of CAD patients. Therefore, the purpose of this study was to investigate the effect of LC supplementation (1000 mg/d) on lipid levels in CAD patients.

## Methods

### Study design

**Fig. 1:**
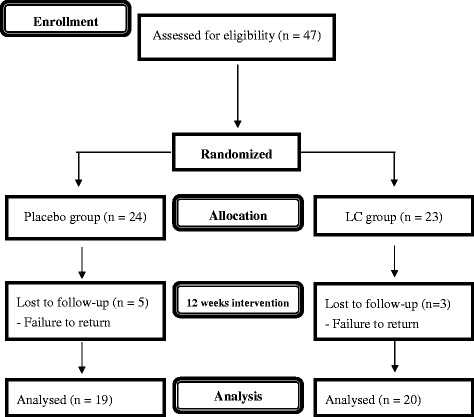
Consort flow diagram

A single-blind, randomized, parallel, placebo-controlled trial was conducted. CAD patients were diagnosed by cardiac catheterization (at least 50 % stenosis of one major coronary artery) or by percutaneous transluminal coronary angioplasty (PTCA) for stable condition. We excluded the patients with diabetes, liver, or renal diseases, along with those undergoing acenocoumarol, thyroid hormone, warfarin, and a high dose of statin therapy [Atorvastatin (Lipitor) or Rosuvastatin (Crestor), 30-40 mg/d] or currently using vitamin supplements. The study was approved by the Institutional Review Board of Taichung Veterans General Hospital, Taiwan and the clinical trial was registered at Clinical Trials.gov (NCT01819701). This clinical trial started recruiting subjects at January 2013, and the last subject was completed in February 2014. Each subject provided written informed consent to participate in the study. A total of 47 CAD patients were recruited to this study and randomly assigned to the placebo (*n* = 24) or to the LC (1000 mg/day, *n* = 23) group. The supplements, placebo (starch) and LC capsules were provided by New Health Taiwan Co., Ltd (Taichung, Taiwan). At the beginning of the study, the investigators instructed the subjects to take two capsules daily (LC supplements 500 mg b.i.d). To monitor the subject compliance, we asked the subjects to return the supplied bag of capsules every 4 weeks to verify the capsule count. The intervention was administered for 12 weeks. The sampling and trial profiles along with the number of subjects who completed the study in each group are presented in Fig. 1.

At baseline, we measured the subjects’ blood pressure, body weight, height, and waist circumference, and then calculated the body mass index (kg/m^2^). General obesity was defined as body mass index ≥ 27 kg/m^2^ [23], and central obesity was defined as waist circumferences ≥ 90 cm. Dietary intakes were assessed using 24-h recall dietary records at baseline. The dietary records were analyzed using the Nutritionist Professional software package (E-Kitchen Business Corp., Taiwan).

### Lipid profiles, L-carnitine, and antioxidant enzyme activity measurements

The fasting blood specimens were collected in vacutainer tubes without anticoagulant (Becton Dickinson, Rutherford, NJ, USA). The samples were centrifuged at 3,000 rpm for 15 minutes at 4 °C, and the serum was separated. Serum total cholesterol (TC), triacylglycerol (TG), low density lipoprotein-cholesterol (LDL-C), and high density lipoprotein-cholesterol (HDL-C) were measured using an automated biochemical analyzer (Hitachi-7180E, Tokyo, Japan). The levels of apolipoprotein A-1 (Apo-A1) and apolipoprotein-B (Apo-B) were measured using the polyethylene glycerol (PEG) enhanced immunoturbidimetric assay method (Siemens Healthcare Diagnostics Inc., New York, USA). The level of LC was measured by the enzyme-linked immunosorbent assay (ELISA) method using commercially available kits (Cusabio, Wuhan, China) according to the instructions made available by the suppliers.

The antioxidant enzyme activity (superoxide dismutase, SOD) were measured in the fresh red blood cells (RBCs) samples. The RBCs were washed with normal saline after removing the plasma, and then diluted with 25x sodium phosphate buffer for measurement. The methods for measuring SOD activity have been described previously [24]. The protein content of the plasma and RBCs was determined based on the biuret reaction of the bicinchoninic acid assay (BCA kit, Thermo, Rockford, IL, USA). SOD values were expressed as U/mg of protein. All of the analyses were performed in duplicate.

### Statistical analysis

Means and standard deviations (s.d.) were calculated for all data. Kolmogorov-Smirnov test was used to examine the normal distribution of variables. Student’s t-test or the Mann-Whitney rank sum test was used to compare mean values for continuous variables between the placebo and LC groups. Paired *t*-test or Wilcoxon signed rank test was used to compare the data before and after supplementation within the group. For categorical response variables, differences between the two groups were assessed by the Chi-square test or Fisher's exact test. Simple linear regressions were used to examine the correlations between the levels of LC (as a dependent variable) and lipid profiles after supplementation. Pearson correlations were used to examine the correlations between the antioxidant enzyme activity and lipid profiles after LC supplementation and the correlations between the changes (week 12-0, △) in antioxidant enzyme activity and lipid profiles. Statistical significance was set at *P* < 0.05. All statistical analyses were performed using SigmaPlot software (version 12.0, Systat, San Jose, CA, USA).

## Results

### Characteristics of the subjects at baseline

A total of 39 CAD subjects completed the 12-week interventional study (Placebo, *n* = 19; LC, *n* = 20). The baseline characteristics of the subjects are shown in Table 1. The means and s.d. for age, blood pressure, and dietary intakes as well as the percentages of general obesity and central obesity were not significantly different between the two groups at baseline.

**Table 1:** Baseline characteristics of the subjects

	Placebo (*n* = 19)		LC (*n* = 20)	
	*Mean*	*s.d.*	*Mean*	*s.d*
Gender (male)	19		20	
Age (y)	72.7	10.1	71.9	10.6
SBP (mmHg)	127.3	6.0	128.4	10.4
DBP (mmHg)	74.5	4.4	72.3	4.7
General obesity^a^ (%)	36.8		30.0	
Central obesity^a^ (%)	84.2		65	
Smoking^b^ (n, %)	3 (15.8 %)		4 (20.0 %)	
Drinking^c^ (n, %)	3 (15.8 %)		3 (15.0 %)	
Dietary intake				
Energy (kcal/day)	1501.4	63.7	1534.2	41.6
Protein (g/day, % of total calories)	54.2 (14.2)	11.2	51.1 (12.9)	6.9
Fat (g/day, % of total calories)	43.3 (26.4)	9.7	43.2 (23.8)	10.6
SFA (g/day)	11.7	5.3	10.6	5.4
PUFA (g/day)	14.0	3.5	15.9	3.6
MUFA (g/day)	13.3	3.8	13.0	4.9
Carbohydrate (g/day, % of total calories)	225.3 (60.3)	16.3	236.7 (63.5)	24.7

^a^General obesity was defined as body mass index ≥ 27 kg/m^2^ and central obesity was defined as waist circumferences ≥ 90 cm. ^b^Smoking: the individual currently smokes one or more cigarettes per day. ^c^Drinking: the individual regularly drinks one or more drinks per day

*DBP* diastolic blood pressure; *LC* L-carnitine; *MUFA* monounsaturated fatty acids; *PUFA* polyunsaturated fatty acids; *SBP* systolic blood pressure; *SFA* saturated fatty acids

### Effects of LC supplementation on the levels of LC, SOD activity, and lipid profiles

**Fig. 2:**
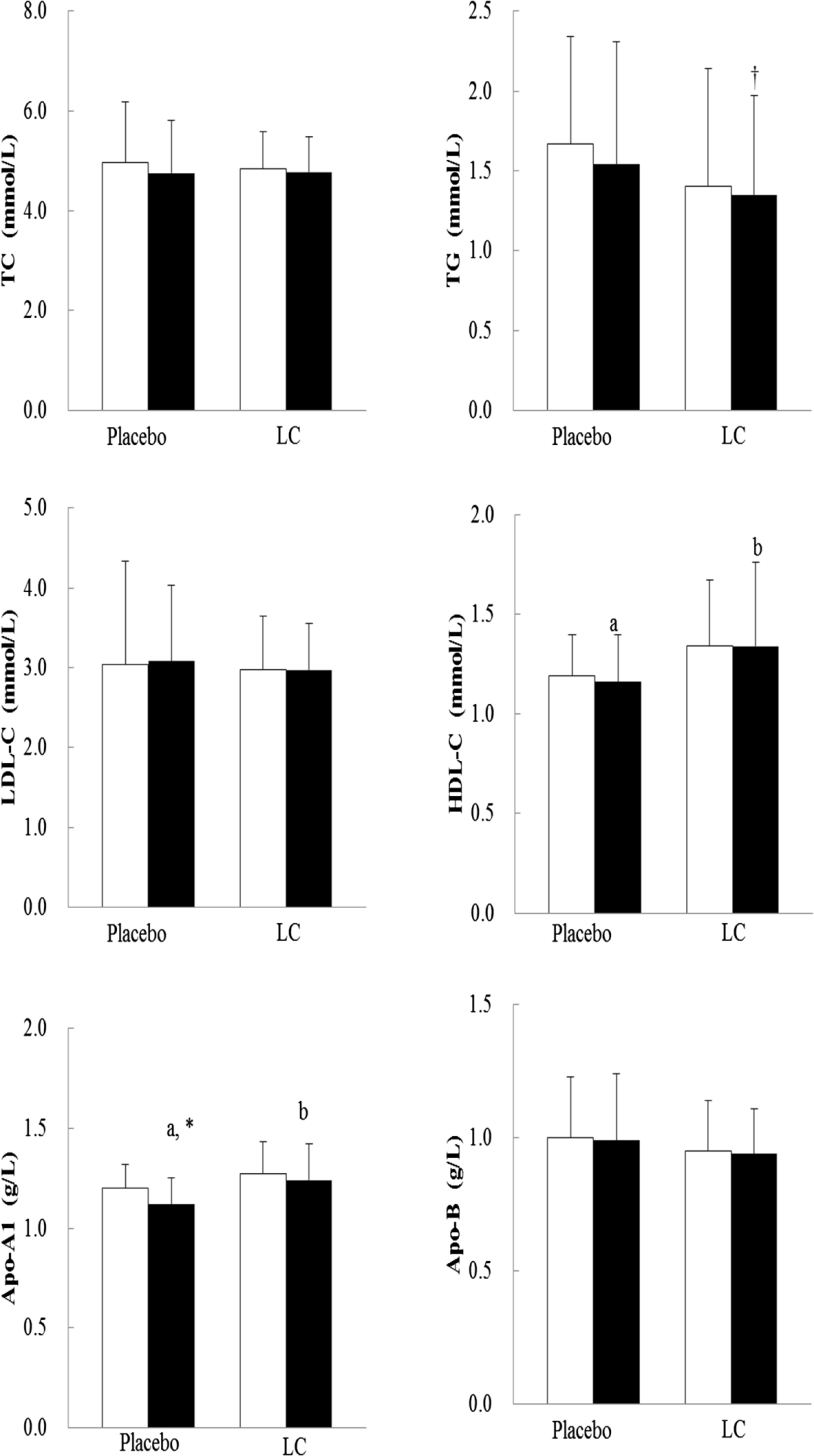
Levels of lipids profiles after supplementation

The subjects in the LC group had significantly increased LC level (40.0 ± 12.0 versus.35.2 ± 12.0 μmol/L, *P* = 0.02) and SOD activity (20.7 ± 4.2 versus 13.1 ± 2.9 U/mg of protein, *P* < 0.01) compared with those in the placebo group at week 12 as well as after LC supplementation (LC, 33.6 ± 13.6 to 40.0 ± 12.0 μmol/L, *P* = 0.04; SOD, 14.8 ± 2.9 to 20.7 ± 4.2 U/mg of protein, *P* < 0.01). The lipid profiles after supplementation are shown in Fig. 2. The subjects in the LC group had significantly higher level of HDL-C (1.34 ± 0.42 vs. 1.16 ± 0.24 mmol/L, *P* = 0.03) and Apo-A1 (1.24 ± 0.18 vs. 1.12 ± 0.13 g/L, *P* = 0.02) than those in the placebo group at week 12. After LC supplementation, the level of TG was slightly reduced but did not achieve statistical significance (1.40 ± 0.74 vs. 1.35 ± 0.62 mmol/L, *P* = 0.06, respectively). However, no significant change in the levels of TC, LDL-C, and Apo-B after 12 weeks of LC supplementation. Notably, the level of Apo-A1 was decreased in the placebo group after 12 weeks intervention (1.20 ± 0.12 vs. 1.12 ± 0.13 g/L, *P* = 0.05); however, the result was not statistically significant.

### Correlations between the levels of lipid profiles and LC after supplementation

**Fig. 3:**
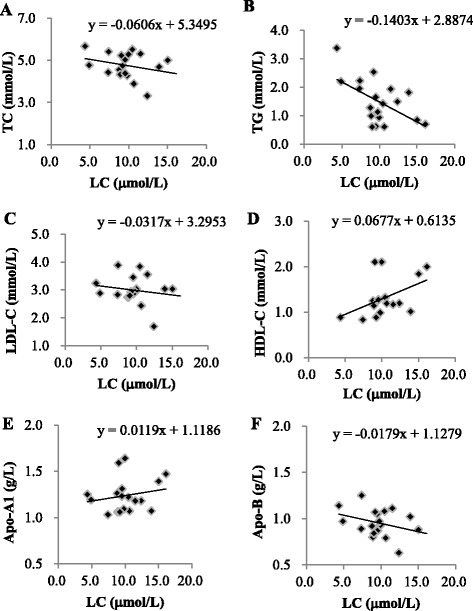
Correlations between the levels of lipids profiles and L-carnitine after supplementation

The correlations between the levels of lipid profiles and LC after supplementation and these results are shown in Fig. 3. The level of LC was significantly negatively correlated with TG (*β* = -0.14, *P* = 0.01). Although not statistically significant, LC level correlated with Apo-B (*β* = -0.02, *P* = 0.08), HDL-C (*β* = 0.07, *P* = 0.07) and Apo-A1 (*β* = 0.01, *P* = 0.09) after 12 weeks of supplementation.

### Correlations between the SOD activity and lipid profiles after LC supplementation

The correlations between the antioxidant enzyme activity and lipid profiles after LC supplementation are shown in Table 2. SOD activity was significantly negatively correlated with TC (*r* = -0.34, *P* = 0.03), TG (*r* = -0.53, *P* < 0.01), and Apo-B (*r* = -0.32, *P* < 0.05) after 12 weeks of LC supplementation. Furthermore, we assessed the correlations between the changes (week 12-0, △) in antioxidant enzyme activity and lipid profiles (Table 3). There was a significant correlation between the changes level of SOD activity and the changes in TC (*r* = -0.41, *P* < 0.01), LDL-C (*r* = -0.45, *P* < 0.01), and Apo-B (*r* = -0.32, *P* < 0.01).

**Table 2:** Correlations between the antioxidant enzyme activity and lipid profiles after L-carnitine supplementation

	SOD (U/mg protein)
	*r* ^a^ (*P* values)
TC (mmol/L)	−0.34 (0.03)
TG (mmol/L)	−0.53 (< 0.01)
LDL-C (mmol/L)	−0.15 (0.36)
HDL-C (mmol/L)	0.11 (0.52)
Apo-A1 (g/L)	−0.18 (0.29)
Apo-B (g/L)	−0.32 (< 0.05)

^a^correlation coefficients

*Apo* apolipoprotein; *HDL-C* high density lipoprotein-cholesterol; *LC* L-carnitine; *LDL-C* low density lipoprotein-cholesterol; *SOD* superoxide dismutase; *TC* total cholesterol; *TG* triglycerides

**Table 3:** Correlations between the changes (week 12-0, △) in antioxidant enzyme activity and lipid profiles

	△SOD (U/mg protein)
	*r* ^a^ (*P* values)
△TC (mmol/L)	−0.41 (< 0.01)
△TG (mmol/L)	−0.11 (0.34)
△LDL-C (mmol/L)	−0.45 (< 0.01)
△HDL-C (mmol/L)	−0.04 (0.77)
△Apo-A1 (g/L)	−0.09 (0.44)
△Apo-B (g/L)	−0.32 (< 0.01)

^a^correlation coefficients

*Apo* apolipoprotein; *HDL-C* high density lipoprotein-cholesterol; *LC* L-carnitine; *LDL-C* low density lipoprotein-cholesterol; *SOD* superoxide dismutase; *TC* total cholesterol; *TG* triglycerides

## Discussion

Significant lipid-lowering effects of LC supplementation have been demonstrated in hemodialysis patients [16, 17]. Naini et al. treated chronic hemodialysis patients with oral LC supplementation at doses of 750 mg/d and 1000 mg/d for 8 weeks and 16 weeks, respectively. LC supplementation at a dose of 750 mg/d significantly decreased levels of lipid (TC, TG, and LDL-C) [17], whereas LC supplementation at a dose of 1000 mg/d significantly decreased levels of TC and TG and increased the level of HDL-C [16]. LC may increase the mitochondrial transport of fatty acids and reduce fatty acid availability for lipid synthesis [25]. LC has also improved the levels of lipid in CAD given that LC is a lipid transporter. In this study, we observed that LC administered at a dose of 1000 mg/d for 12 weeks correlated with decreased lipid profile (TG) levels and increased levels of HDL-C and Apo-A1. Our previous studies demonstrated that LC supplementation at a dose of 1000 mg/d was significantly associated with increased antioxidant capacity in CAD patients [26]. In addition, the level of LC was significantly positively correlated with antioxidant enzymes activity (SOD, *β* = 0.72, *P* < 0.01) and reduced oxidative stress and inflammatory status in the patients with CAD after supplementation [26, 27]. In this study, we observed no significant correlation between LC and lipids levels or between SOD activity and lipid levels at baseline. Instead, antioxidant enzyme activity (SOD) was significantly negatively correlated with lipid levels (TC, TG, and Apo-B) after LC supplementation (Table 2). As a result, wehypothesize that the effect of LC supplementation on lipids (TG, HDL-C, and Apo-A1) may be related to its antioxidant capacity.

Apolipoproteins, such as Apo-B and Apo A-1, are the major apolipoproteins associated with LDL-C and HDL-C, respectively [28]. In this study, we observed a significant increase in the levels of Apo-A1 and HDL-C, and the level of LC was slightly negatively correlated with Apo-B after supplementation. However, no significant change in the levels of TC and LDL-C were noted after LC supplementation. In a clinical study, Malaguarnera et al. investigated diabetes patients using a higher dose of LC supplementation of 2000 mg/d for 12 weeks and observed significant decreases in the levels of TC, TG, LDL-C, oxidized LDL-C, and Apo-B, and increases in the levels of HDL-C and Apo-A1 [20]. Hyperlipidemia is a contributor to CAD; thus, we considered that if the disease has already developed, the lipid-lowering effect of LC supplementation at a dose of 1000 mg/d may not be sufficient. Although LC at a dose of 1000 mg/d can significantly increase the antioxidant activity [26], a higher dose of LC (>1000 mg/d) administration could be considered in anti-hyperlipidemic strategies [29, 30].

In the present study, TG level decreased after supplementation, but the reduction did not achieve statistical significance, which may have been attributed to the stable CAD subjects and the limited number of subjects (Placebo, *n* = 13; LC, *n* = 8) with hyperlipidemia (a ratio of TC to HDL ≥ 5.0, TG ≥ 1.7 mmol/L, or LDL-C ≥ 3.4 mmol/L) [31]. Due to the small number of subjects having hyperlipidemia in the LC group, the detection of the lipid-lowering effects in these subjects may have been limited. Because higher oxidative stress and inflammation status are early events in the evolution of hyperlipidemia [32, 33], we suggest that an antioxidant supply (such as LC) may help prevent or delay the development of CAD [26, 27]. The lipid-lowering effects of a clinically relevant degree of LC supplementation might be related to its dosage. Further studies should be conducted to define an optimal dose of LC for lipid-lowering in patients with CAD and explore whether it could be used as a nutraceutical adjuvant in the cases of dyslipidemia [34].

The strength of this study is that it is the first clinical study to investigate the lipid-lowering effects of LC supplementation in CAD patients, and we measured the levels of LC, lipid profiles, and antioxidant enzyme activity. This study provides direct evidence to clarify the relationship among the levels of LC, lipid, and antioxidant ability; however, longer intervention studies with larger sample sizes should be performed to confirm the lipid-lowering effects after LC intervention in patients with hyperlipidemia. There are some limitations of the present study that should be mentioned. First, the number of participants was small; however, we performed the post hos calculations to examine the statistical power for lipid profiles. The statistical power of the differences of TG, HDL-C, and Apo-A1 were 0.97, 0.86, and 0.86, respectively. Second, a few of the subjects had hyperlipidemia in the LC group; thus, the detection of the lipid-lowering effects in these subjects may have been limited. Moreover, an optimal dose of LC for lipid-lowering should be defined. Further studies should also investigate the effect of LC on NADPH oxidase activity, HMG-CoA reductase activity and TG synthesis to clarify if LC lowers lipids by enhancing mitochondrial fatty acid oxidation or by reducing oxidative stress.

## Conclusion

In conclusion, LC supplementation at a dose of 1000 mg/d exhibited significant increases in HDL-C and Apo-A1 levels and a slight decrease in TG levels, but no other changes in other lipids were noted in CAD patients. This lipid-lowering effect may be related to its antioxidant ability.
